# Activation of Toll-like receptor 2 enhances peripheral and tumor-infiltrating CD8^+^ T cell cytotoxicity in patients with gastric cancer

**DOI:** 10.1186/s12865-021-00459-z

**Published:** 2021-10-07

**Authors:** Junli Xu, Rongya Guo, Jing Jia, Yun He, Shuixiang He

**Affiliations:** 1 Department of Gastroenterology, The First Affiliated Hospital of Xi’an Jiaotong University, No. 277 Yanta West Rd, Xi’an, 710061 Shaanxi Province China; 2 Department of Gastroenterology, Xi’an No.1 Hospital, Xi’an, 710002 Shaanxi Province China; 3 Department of Chemistry, Shaanxi Institute for Food and Drug Control, Xi’an, 710065 Shaanxi Province China; 4 Department of Dermatology, Xi’an No.1 Hospital, Xi’an, 710002 Shaanxi Province China

**Keywords:** Gastric cancer, Toll-like receptor, CD8^+^ T cells, Immunomodulation

## Abstract

**Background:**

Toll-like receptors (TLRs) play central roles in the initiation of innate immune response, and also control adaptive immunity activation. Thus, the aim of the study was to investigate the regulation of TLR activation to CD8^+^ T cells has not been fully elucidated in gastric cancer (GC).

**Materials and methods:**

Thirty-two GC patients and twenty-three healthy controls were enrolled. Expression profile of TLRs in peripheral and tumor-infiltrating CD8^+^ T cells was investigated. Purified CD8^+^ T cells were stimulated with Pam3Csk4, an agonist of TLR2, and cytotoxic and co-inhibitory molecules in CD8^+^ T cells was measured. Direct and indirect contact coculture system between CD8^+^ T cells and AGS cells was set up. Modulation of TLR2 activation to CD8^+^ T cells was assessed by measuring lactate dehydrogenase release and cytokine secretion.

**Results:**

TLR2 mRNA and TLR2^+^ cell percentage was down-regulated in GC derived peripheral and tumor-infiltrating CD8^+^ T cells. CD8^+^ T cells from GC patients showed exhausted phenotype, which presented as decreased perforin/granzyme B, increased programmed death-1, and reduced cytotoxicity to AGS cells. TLR2 activation by Pam3Csk4 enhanced perforin and granzyme B expression in CD8^+^ T cells, however, did not affect either proinflammatory cytokine production or co-inhibitory molecules expression. Pam3Csk4 stimulation enhanced cytolytic activation of peripheral and tumor-infiltrating CD8^+^ T cells from GC, but not those from healthy individuals.

**Conclusion:**

The present data revealed an important immunomodulatory activity of TLR2 to CD8^+^ T cells in GC patients.

## Background

Gastric cancer (GC) is still one of the major public health problems, ranking the third leading cause of cancer-related mortality all over the world [1]. The diagnosis and therapeutic strategy for GC have been improved, however, the 5-year overall survival rate of GC remains poor due to extensive invasion and metastasis [2]. Thus, it is still of great importance to develop new therapeutic approaches for GC patients. Gastrointestinal tract has been proven to be a highly specialized immune environment, and increasing numbers of immune cells, molecules, and signaling pathways play vital roles in gastrointestinal homeostasis [3]. Unfortunately, evolution and progression of GC always facilitates the exhaustion and dysfunction of immune system, leading to the losing in effector functions and insufficient control of malignancy [4]. Therefore, several preclinical and clinical experiences using immunoconjugates provide new insights for GC therapy [5, 6].

Toll-like receptors (TLRs) are important sensors in innate immune system to a wide variety of molecules, such as invading microorganisms and internal damaged tissues [7]. TLRs are expressed in both immune cells (including antigen presenting cell, monocytes, marcophages, and T cells) and parenchymal cells (such as tumor cells and neurons). Activation of TLRs induces the elevation of various cytokines and chemokines expression, which controls the adaptive immunity [8]. In tumor microenvironment, TLRs activation plays bio-functional activity with antitumor (activation of dendritic cells, cytotoxic T cells, and natural killer cells) [9, 10] and protumor effects (proliferation and survival of tumor cells, resistance to chemotherapy, and drivers of tumorigenesis) [11]. However, the role of TLRs in regulation of adaptive immune response has not been fully elucidated in GC patients. It has been shown that TLRs signals licensed CD8^+^ T cell effector functions in both physiological and pathological condition [12–15]. Thus, we hypothesized that TLR signaling pathway activation also modulated the activity of peripheral and tumor-infiltrating CD8^+^ T cells in GC patients. To test this possibility, we examined the expression profile of TLRs in CD8^+^ T cells in GC patients, and assessed the regulatory role of TLR agonists to cytolytic and non-cytolytic function of purified CD8^+^ T cells in vitro.

## Materials and methods

### Subjects

The study was conformed to the ethical guidelines of the 1975 Declaration of Helsinki. The protocol was approved by the Institutional Review Board and Ethics Committee of The First Affiliated Hospital of Xi’an Jiaotong University on March 2018, and written informed consent was obtained from each participant. Thirty-two GC patients, who were hospitalized in The First Affiliated Hospital of Xi’an Jiaotong University between July 2018 and February 2019, were enrolled in this study. Patients who received chemotherapy or immunomodulatory therapy before baseline sampling were excluded from the study. No patients were concurrently afflicted by autoimmune diseases, chronic infections, organ failure, or other cancers. Twenty-three healthy individuals with matched age and sex ratio were also enrolled as normal control (NC). The baseline data of enrolled subjects were shown in Table 1.

**Table 1:** Baseline data of enrolled subjects

Variables	GC patients (*n* = 32)	NC (*n* = 23)
Age (year)^#^	47 (28 ~ 61)	48 (33 ~ 62)
Gender (male/female)	23/9	13/10
Helicobacter pylori infection (*n*)	29	11
TNM staging (I/II/III/IV)	7/13/7/5	Not available
Lauren classification (diffuse/intestinal/mixed)	15/7/10	Not available

^#^Data were presented as median with a range

### Peripheral blood mononuclear cells (PBMC) and tumor-infiltrating lymphocytes (TIL) isolation

Twenty milliliters of ethylenediaminetetraacetic acid anti-coagulant peripheral bloods were obtained from each enrolled subjects. PBMCs were isolated using Ficoll-Hypaque (Solarbio, Beijing, China) density-gradient centrifugation. TILs were purified from tumor samples, which were obtained from GC patients who were underwent surgery. Tumor specimens were cut into small pieces, and were passed through 70 μm-pore strainers. Cells were treated with Collagenase D (0.5 mg/ml) at 37 °C for 30 min, followed by resuspension in 44% Percoll in RPMI 1640 (*vol/vol*). Cells were then layered over 56% Percoll in PBS (*vol/vol*), and were centrifuged at 850*r*/min for 30 min. The interphase, which contained TILs, was collected. PBMCs and TILs were incubated at 37 °C in a humidified atmosphere containing 5% CO_2_, and cultured in RPMI 1640 combined with 10% fetal bovine serum (FBS) at a concentration of 10^6^/ml.

### Enrichment of CD8^+^ T cells

CD8^+^ T cells were enriched using human CD8^+^ T Cell Isolation Kit (Miltenyi, Bergisch Gladbach, Germany). The purity of enriched cells was more than 95% based on flow cytometry determination.

### Cell culture and treatment

Human GC cell line AGS, which were confirmed as HLA-A*0201 restricted [16], were purchased from the Cell Bank of Chinese Academy of Sciences. AGS cells were incubated at 37 °C in a humidified atmosphere containing 5% CO_2_, and cultured in RPMI 1640 combined with 10% FBS. Purified CD8^+^ T cells were stimulated with Pam3Csk4 (InvivoGen, San Diego, CA, USA; 400 ng/ml) for 12 h, and were washed twice. 10^5^ of Pam3Csk4-stimulated CD8^+^ T cells from HLA-A2-restricted subjects were cocultured in direct contact or in indirect contact with 5 × 10^5^ of AGS cells in the presence of anti-CD3/CD28 (eBioscience, San Diego, CA). In direct contact coculture system, CD8^+^ T cells and AGS cells were directly mixed for culture. In indirect contact coculture system, CD8^+^ T cells and AGS cells were separated by a 0.4 μm-pore membrane in a Transwell culture plate insert (Corning, Corning, NY), which only allowed the passage of soluble factors [17–20]. Supernatants were harvested 48 h post coculture.

### Real-time polymerase chain reaction (PCR)

Total RNA was isolated from 10^5^ of purified CD8^+^ T cells using Trizol Reagent (Invitrogen, Carlsbad, CA) following manufacturer’s instruction. First-strand cDNA was obtained by reverse transcription from 1 μg of total RNA using PrimeScript RT Reagent Kit (TaKaRa, Beijing, China). Real-time PCR was performed using TB Green Premix Ex *Taq* (TaKaRa) following manufacturer’s instruction. Relative gene expression was semi-quantified by 2^*−ΔΔCT*^ method using 7500 System Sequence Detection Software (Applied Biosystems, Foster, CA). The sequences of primers and amplification program were described previously [21].

### Flow cytometry

Cells were stained with anti-CD3-PerCP Cy5.5 (eBioscience), anti-CD8-APC (BD Bioscience, San Jose, CA), anti-TLR2-PE (eBioscience), anti-TLR7-FITC (eBioscience), anti-lymphocyte activation gene-3 (LAG-3)-PerCP (BD Bioscience), and anti-CD279 (programmed death-1, PD-1)-FITC (BD Bioscience) following manufacturer’s instructions. Acquisitions were performed using CellQuest Pro Software by FACS Calibur (BD Bioscience), and data were analyzed using FlowJo V10 (TreeStar, Ashland, OR).

### Enzyme-linked immunosorbent assay (ELISA)

Cytokine expression was measured using commercial ELISA kits (CUSABio, Wuhan, Hubei Province, China).

### Enzyme-linked immunospot assay (ELISPOT)

Perforin and granzyme B secretion by CD8^+^ T cells was measured using Human Perforin ELISPOT Kit (Abcam, Cambridge, MA) and Human Granzyme B ELISPOT Kit (Abcam).

### Cytotoxicity assay

The cytotoxicity of AGS cells were assessed by measurement of lactate dehydrogenase (LDH) expression in the cultured supernatants using Cytotoxicity Assay Kit (Beyotime, Wuhan, Hubei Province, China). The low level control was defined as LDH expression in the supernatant of AGS cells, while the high level control was defined as LDH expression in the supernatant of Triton X-100 treated AGS cells. The percentage of target cell death was calculated using the following equation: (experiment value − low level control)/(high level control − low level control) × 100% [17–20].

### Statistical analysis

Data were analyzed using SPSS 21.0 (SPSS, Chicago, IL). Variables were analyzed for normal distribution using Shapiro–Wilk test. Data following normal distribution were presented as mean ± standard deviation (SD), and were analyzed using Student *t* test, paired *t* test, One-Way ANOVA, or LSD-*t* test. Data following skewed distribution were presented as median [Q_1_, Q_3_], and were analyzed using Mann–Whitney test, Wilcoxon matched pairs test, Kruskal–Wallis test, or Dunns multiple comparisons test. All tests were two-tails, and a *P* value less than 0.05 was considered as a significant difference.

## Results

### Down-regulation of TLR2 in CD8^+^ T cells in GC patients

**Fig. 1:**
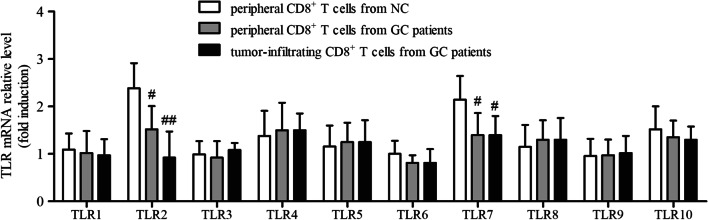
Expression profile of TLRs mRNA in CD8^+^ T cells in GC patients. Peripheral CD8^+^ T cells were purified from NC (*n* = 23) and GC patients (*n* = 32), while tumor-infiltrating CD8^+^ T cells were isolated from GC patients who underwent surgery (*n* = 21). mRNA relative level corresponding to TLR1 ~ TLR10 in CD8^+^ T cells was semi-quantified by real-time PCR, and was compared among peripheral bloods of NCs, peripheral bloods of GC patients, and tumor-residency of GC patients. The columns indicated means, and the bars indicated standard deviation. Significances were determined by One-Way ANOVA, LSD-*t* test, or Kruskal–Wallis test. ^#^*P* < 0.05 compared with NC. ^##^*P* < 0.01 compared with NC

**Fig. 2:**
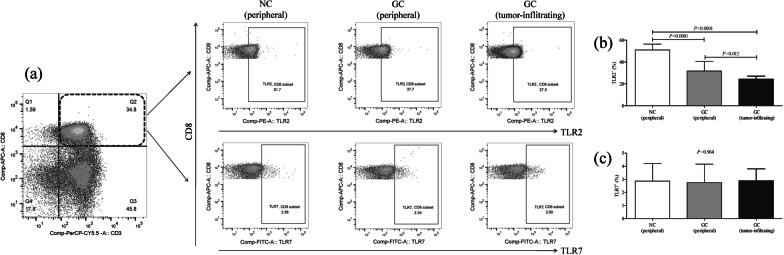
Expression profile of TLR2 and TLR7 protein level in CD8^+^ T cells from peripheral bloods of NCs (*n* = 23), peripheral bloods of GC patients (*n* = 32), and tumor-residency of GC patients who underwent surgery (*n* = 21). Cells were stained with anti-CD3, anti-CD8, anti-TLR2, and anti-TLR7. The percentage of TLR2 and TLR7 positive cells within CD3^+^CD8^+^ T cells was analyzed by flow cytometry. **a** The representative flow dots for TLR2^+^ and TLR7^+^ cells within CD3^+^CD8^+^ T cells were shown. The percentage of **b** TLR2^+^ and **c** TLR7^+^ cells within CD3^+^CD8^+^ T cells was compared peripheral bloods of NCs, peripheral bloods of GC patients, and tumor-residency of GC patients. The columns indicated means, and the bars indicated standard deviation. Significances were determined by One-Way ANOVA and LSD-*t* test

We firstly screened the expression profile of TLRs in CD8^+^ T cells in GC patients. Peripheral CD8^+^ T cells were isolated from all enrolled subjects, while tumor-infiltrating CD8^+^ T cells were purified from twenty-one GC patients who underwent surgery. mRNA expression corresponding to TLR1 ~ 10 in CD8^+^ T cells was semi-quantified by real-time PCR. There were no significant differences of TLR1, TLR3, TLR4, TLR5, TLR6, TLR8, TLR9, or TLR10 mRNA relative level in CD8^+^ T cells among peripheral bloods of NCs, peripheral bloods of GC patients, and tumor-residency of GC patients (One-Way ANOVA or Kruskal–Wallis test, all *P* > 0.05, Fig. 1). TLR7 mRNA relative level in CD8^+^ T cells from GC patients (both peripheral and tumor-infiltrating) was approximate twofold reduced in comparison with that in CD8^+^ T cells from NCs (LSD-*t* test, *P* < 0.05, Fig. 1). mRNA expression of TLR2 in tumor-infiltrating CD8^+^ T cells was remarkable decreased in comparison with that in peripheral CD8^+^ T cells from NCs (LSD-*t* test, *P* < 0.01, Fig. 1) and from GC patients (LSD-*t* test, *P* < 0.05, Fig. 1). Moreover, the percentage of TLR2^+^ and TLR7^+^ cells within CD3^+^CD8^+^ T cells was analyzed by flow cytometry. The representative flow dots for TLR2 and TLR7 expression in CD3^+^CD8^+^ T cells were shown in Fig. 2a. The percentage of TLR2^+^ cells within CD3^+^CD8^+^ T cells in peripheral bloods (31.67 ± 8.84%) and tumor residency (24.37 ± 2.72%) of GC patients was notably lower than in peripheral bloods of NCs (50.96 ± 5.45%) (LSD-*t* tests, *P* < 0.0001, Fig. 2b). However, there was no remarkable difference of TLR7^+^ cells within CD3 + CD8 + T cells among three groups (One-Way ANOVA, *P* = 0.964, Fig. 2c).

### TLR2 activation elevated perforin and granzyme B expression in CD8^+^ T cells

**Fig. 3:**
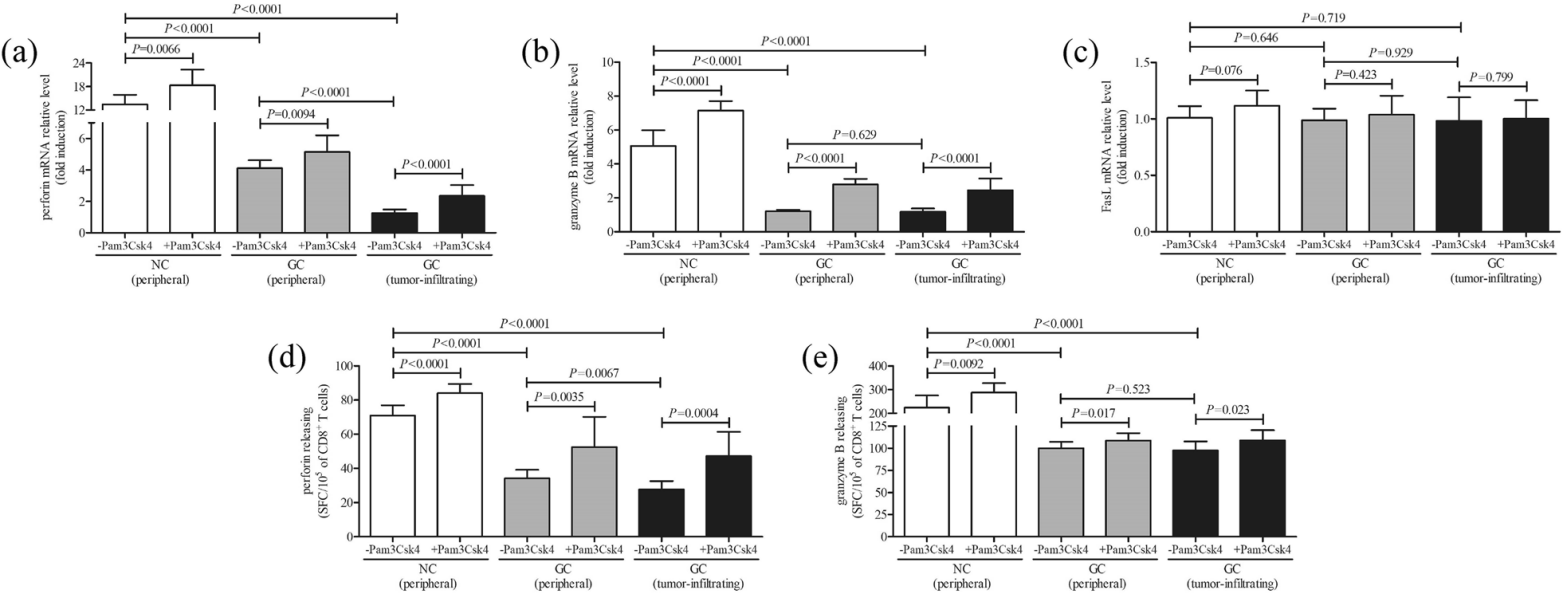
The influence of TLR2 activation on cytotoxic pathways of peripheral and tumor-infiltrating CD8^+^ T cells from NC and GC patients. CD8^+^ T cells were purified from peripheral bloods of NC (*n* = 9) and GC patients (*n* = 11), as well as from tumor residency of GC patients (*n* = 11). 2.5 × 10^4^ of CD8^+^ T cells were cultured for 12 h in the presence of anti-CD3/CD28 with or without TLR2 agonist Pam3Csk4. mRNA relative level corresponding to **a** perforin, **b** granzyme B, and **c** FasL in CD8^+^ T cells with or without PamCsk4 stimulation was semi-quantified by real-time PCR. The secretion of **d** perforin and **e** granzyme B by CD8^+^ T cells was analyzed by ELISPOT. The columns indicated means, and the bars indicated standard deviation. Significances were determined by LSD-*t* test or paired *t* test

2.5 × 10^4^ of CD8^+^ T cells from peripheral bloods of NC (*n* = 9), peripheral bloods of GC patients (*n* = 11), and tumor-residency of GC patients (*n* = 11) were cultured for 12 h in the presence of anti-CD3/CD28 with or without Pam3Csk4, a TLR2 agonist which was used for TLR2 activation. Cytokine production by CD8^+^ T cells in cultured supernatants was investigated by ELISA. Expression of proinflammatory cytokines, including interferon-γ (IFN-γ), tumor necrosis factor-α (TNF-α), interleukin (IL)-2, IL-6, and IL-8, was significantly reduced in cultured CD8^+^ T cells from GC patients when compared with those from NC (Dunns multiple comparisons test or LSD-*t* test, all *P* < 0.01, Table 2). However, there were no remarkable differences of cytokines level by CD8^+^ T cells with or without Pam3Csk4 stimulation (Wilcoxon matched pairs test or paired *t* test, all *P* > 0.05, Table 2). mRNA expression corresponding to perforin, granzyme B, and FasL in CD8^+^ T cells was semi-quantified by real-time PCR. Perforin and granzyme B mRNA relative level in peripheral and tumor-infiltrating CD8^+^ T cells from GC patients was significantly reduced in comparison with those from NC (LSD-*t* test, all *P* < 0.0001, Fig. 3a, b). Importantly, TLR2 activation robustly elevated perforin and granzyme B mRNA expression in CD8^+^ T cells in all three groups (paired *t* test, all *P* < 0.01, Fig. 3a, b). However, FasL mRNA relative level in CD8^+^ T cells was comparable among three groups (LSD-*t* test, *P* > 0.05, Fig. 3c), while TLR2 activation did not influence FasL mRNA expression in CD8^+^ T cells in all three groups (paired *t* test, *P* > 0.05, Fig. 3c). Furthermore, perforin and granzyme B secretion by CD8^+^ T cells was also assessed by ELISPOT. The spot-form cells (SFC)/10^5^ of CD8^+^ T cells of perforin and granzyme B was also remarkably decreased in GC patients (both peripheral and tumor-infiltrating) when compared with in NC (LSD-*t* test, all *P* < 0.0001, Fig. 3d, e). PamCsk4 stimulation notably increased perforin and granzyme B secretion by CD8^+^ T cells in all three groups (paired *t* test, all *P* < 0.05, Fig. 3d, e).

**Table 2:** Cytokine production by CD8^+^ T cells in response to TLR2 activation (pg/ml)

	NC (peripheral) (*n* = 9)		GC (peripheral) (*n* = 11)		GC (tumor-infiltrating) (*n* = 11)	
	− Pam3Csk4	+ Pam3Csk4	− Pam3Csk4	+ Pam3Csk4	− Pam3Csk4	+ Pam3Csk4
IFN-γ	782.2 [243.1, 1107]	778.9 [256.1, 997.0]	621.7 [334.6, 1008]^#^	597.3 [287.1, 1109]	572.9 [231.8, 983.1]^#^	596.1 [229.8, 963.0]
TNF-α	97.14 ± 28.08	98.91 ± 10.95	76.64 ± 20.35^#^	71.90 ± 17.63	66.08 ± 18.24^#^	70.19 ± 23.73
IL-2	108.2 ± 31.39	117.0 ± 39.27	87.09 ± 21.22^#^	90.83 ± 14.93	88.18 ± 10.31^#^	89.07 ± 19.52
IL-6	57.05 ± 13.42	55.19 ± 14.27	39.21 ± 10.98^#^	34.16 ± 9.08	40.13 ± 14.24^#^	45.29 ± 7.93
IL-8	229.4 ± 78.15	241.4 ± 67.63	164.7 ± 40.42^#^	171.3 ± 51.84	170.8 ± 48.33^#^	166.0 ± 45.72

^#^ *P* < 0.01 compared with NC

### TLR2 activation did not affect LAG-3 or PD-1 expression in CD8^+^ T cells

**Fig. 4:**
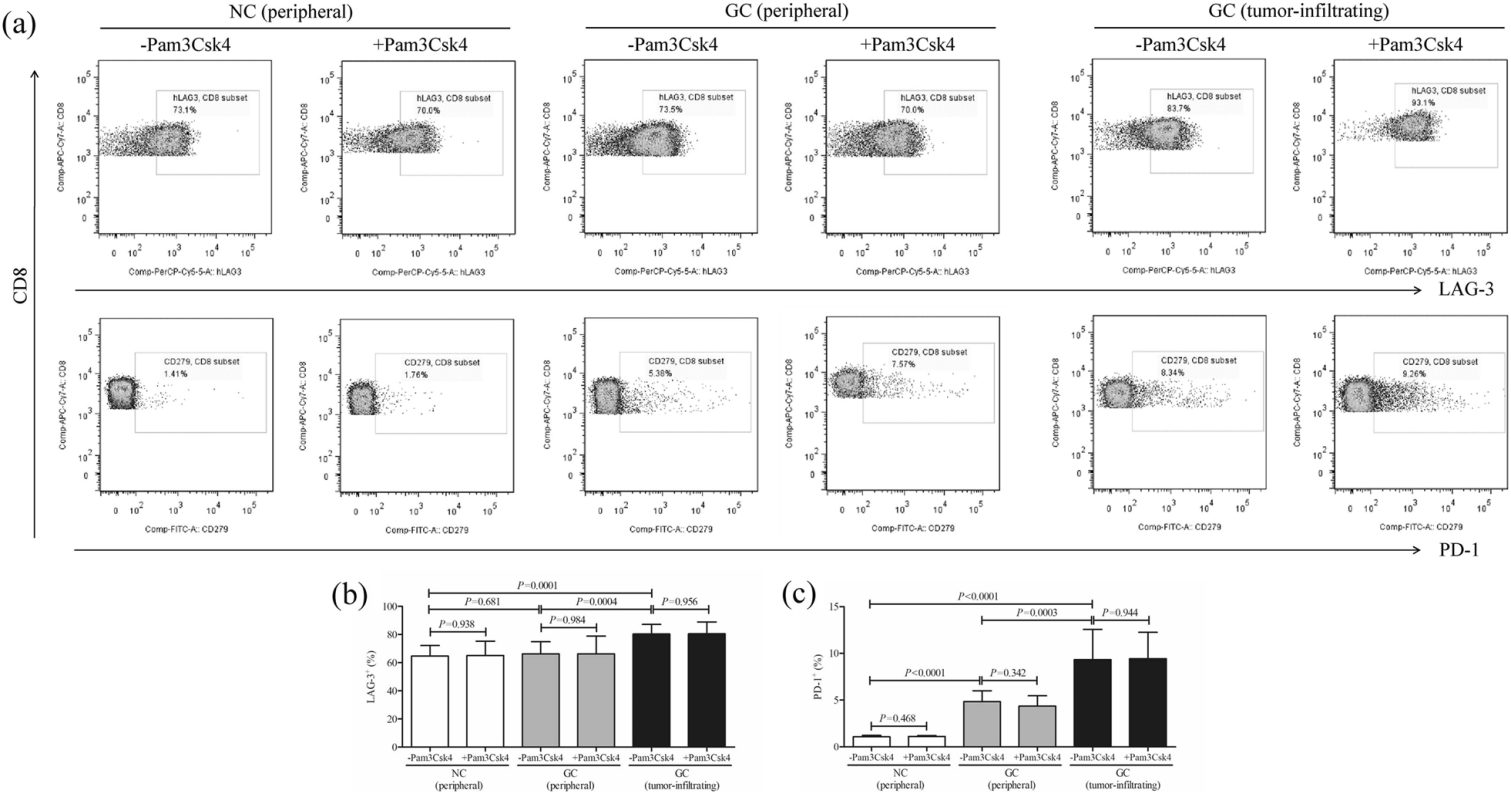
The influence of TLR2 activation on inhibitory molecules expression in peripheral and tumor-infiltrating CD8^+^ T cells from NC and GC patients. CD8^+^ T cells were purified from the same subjects as in Fig. 2, including peripheral bloods of NC (*n* = 9) and GC patients (*n* = 11), as well as from tumor residency of GC patients (*n* = 11), and were cultured for 12 h in the presence of anti-CD3/CD28 with or without TLR2 agonist Pam3Csk4. Lymphocyte activation gene-3 (LAG-3) and CD279 (programmed death-1, PD-1) expression in CD8^+^ T cells was measured by flow cytometry. **a** The representative flow dots of LAG-3 and PD-1 positive cells in peripheral CD8^+^ T cells from NC and GC patients, as well as in tumor-infiltrating CD8^+^ T cells from GC patents with or without Pam3Csk4 stimulation. The percentage of **b** LAG-3^+^ and **c** PD-1^+^ cells within CD8^+^ T cells was compared. The columns indicated means, and the bars indicated standard deviation. Significances were determined by LSD-*t* test or paired *t* test

The inhibitory molecules (LAG-3 and PD-1) expression in CD8^+^ T cells from peripheral bloods of NC (*n* = 9), peripheral bloods of GC patients (*n* = 11), and tumor-residency of GC patients (*n* = 11) was also measured by flow cytometry. The representative flow dots of LAG-3 and PD-1 positive cells in CD8^+^ T cells with or without Pam3Csk4 stimulation were shown in Fig. 4a. The percentage of LAG-3^+^ cells was significantly elevated in tumor-infiltrating CD8^+^ T cells (80.27 ± 6.89%) when compared with peripheral CD8^+^ T cells either from NC (64.67 ± 7.43%, LSD-*t* test, *P* = 0.0001, Fig. 4b) or from GC patients (66.18 ± 8.57%, LSD-*t* test, *P* = 0.0004, Fig. 4b). Moreover, The percentage of PD-1^+^ cells was robustly increased in both peripheral (4.83 ± 1.18%) and tumor-infiltrating CD8^+^ T cells (9.34 ± 3.23%) from GC patients when compared with peripheral CD8^+^ T cells from NC (1.06 ± 0.15%, LSD-*t* test, all *P* < 0.0001, Fig. 4c). However, Pam3Csk4-induced TLR2 activation did not affect either LAG3^+^ or PD-1^+^ cell frequency within CD8^+^ T cells (paired *t* test, all *P* > 0.05, Fig. 4b, c).

### TLR2 activation promoted cytolytic function of CD8^+^ T cells from GC patients

**Fig. 5:**
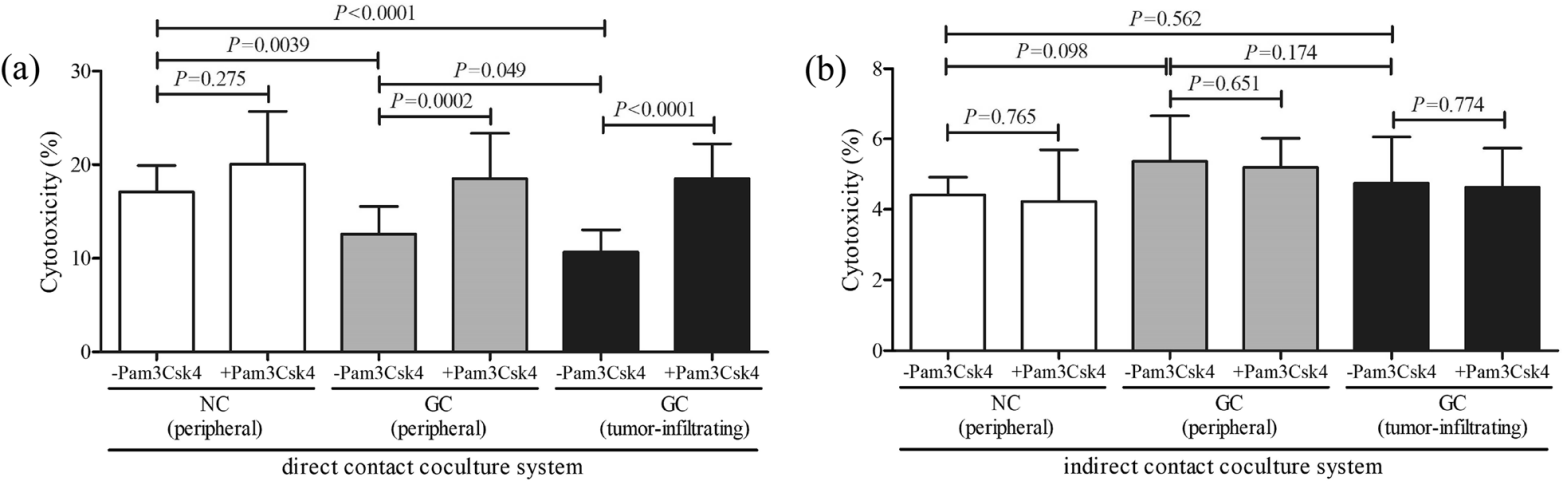
The influence of TLR2 activation on cytolytic function of peripheral and tumor-infiltrating CD8^+^ T cells from NC and GC patients. CD8^+^ T cells were purified from HLA-A2 restricted individuals, including NC (peripheral bloods, *n* = 6) and GC patients (peripheral bloods and tumor specimens, *n* = 17), and were stimulated with or without Pam3Csk4 for 12 h in the presence of anti-CD3/CD28. Cells were washed twice, and 10^5^ of stimulated CD8^+^ T cells were cocultured in direct and indirect contact with 5 × 10^5^ of AGS cells for 48 h. The percentage of AGS cell death in **a** direct contact coculture system and **b** indirect contact coculture system was calculated by measuring lactate dehydrogenase release in the supernatants. The columns indicated means, and the bars indicated standard deviation. Significances were determined by One-Way ANOVA, LSD-*t* test or paired *t* test

CD8^+^ T cells were purified from HLA-A2 restricted individuals, including NC (peripheral bloods, *n* = 6) and GC patients (peripheral bloods and tumor tissues, *n* = 17), and were stimulated with or without Pam3Csk4 for 12 h in the presence of anti-CD3/CD28. 10^5^ of stimulated CD8^+^ T cells were cocultured in direct and indirect contact with 5 × 10^5^ of AGS cells for 48 h. In direct contact coculture system, CD8^+^ T cells from NCs induced elevated target AGS cell death (17.07 ± 2.81%) when compared with those from GC patients (peripheral: 12.56 ± 2.96%, LSD-*t* test, *P* = 0.0039, Fig. 5a; tumor-infiltrating: 12.56 ± 2.96%, LSD-*t* test, *P* < 0.0001, Fig. 5a). TLR2 activation promoted cytolytic activity of both peripheral (18.48 ± 4.86%, paired *t* test, *P* = 0.0002, Fig. 5a) and tumor-infiltrating CD8^+^ T cells (18.48 ± 3.73%, paired *t* test, *P* < 0.0001, Fig. 5a) from GC patients. The Pam3Csk4-treated CD8^+^ T cells from NC also induced increased target cell death, but this elevation failed to achieve statistical difference (paired *t* test, *P* = 0.275, Fig. 5a). In indirect contact coculture system, CD8^+^ T cells-induced target AGS cell death was comparable among groups (One-Way ANOVA, *P* = 0.182, Fig. 5b). However, TLR2 activation by Pam3Csk4 stimulation did not enhance the cytolytic function of CD8^+^ T cells in three groups (paired *t* test, all *P* > 0.05, Fig. 5b). The cytokine production in the supernatants was also measured by ELISA. The proinflammatory cytokines in the cultured supernatants were robustly decreased in CD8^+^ T cells from GC patients in both direct and indirect contact coculture systems (LSD-*t* test, all *P* < 0.05,Tables 3 and 4). However, Pam3Csk4 stimulation to CD8^+^ T cells did not induced elevated cytokines secretion in three groups (paired *t* test, all *P* > 0.05, Tables 3 and 4).

**Table 3:** Cytokine production in direct contact coculture system response to TLR2 activation (pg/ml)

	NC (peripheral) (*n* = 6)		GC (peripheral) (*n* = 17)		GC (tumor-infiltrating) (*n* = 17)	
	− Pam3Csk4	+ Pam3Csk4	− Pam3Csk4	+ Pam3Csk4	− Pam3Csk4	+ Pam3Csk4
IFN-γ	1722 ± 582.7	1810 ± 430.8	1182 ± 297.2^#^	1019 ± 381.1	971.6 ± 101.8 ^#^	1001 ± 342.6
TNF-α	553.1 ± 174.5	549.7 ± 161.9	391.4 ± 87.14^#^	378.0 ± 92.44	360.5 ± 77.25^#^	371.2 ± 82.41
IL-2	221.8 ± 51.04	241.2 ± 48.16	168.4 ± 32.45^#^	157.9 ± 47.79	178.0 ± 45.18^#^	172.9 ± 34.50
IL-6	109.2 ± 23.48	105.4 ± 38.02	71.03 ± 16.46^#^	77.10 ± 19.24	71.42 ± 17.98^#^	67.14 ± 10.57
IL-8	358.0 ± 100.2	335.1 ± 109.4	234.4 ± 87.01^#^	221.9 ± 57.02	228.1 ± 71.03^#^	231.4 ± 66.02

^#^*P* < 0.05 compared with NC

**Table 4:** Cytokine production in indirect contact coculture system response to TLR2 activation (pg/ml)

	NC (peripheral) (*n* = 6)		GC (peripheral) (*n* = 17)		GC (tumor-infiltrating) (*n* = 17)	
	− Pam3Csk4	+ Pam3Csk4	− Pam3Csk4	+ Pam3Csk4	− Pam3Csk4	+ Pam3Csk4
IFN-γ	809.2 ± 113.4	880.4 ± 155.3	537.4 ± 100.3^#^	507.9 ± 90.67	521.7 ± 138.4 ^#^	568.8 ± 99.35
TNF-α	110.3 ± 34.40	102.0 ± 37.79	80.09 ± 11.48^#^	85.75 ± 10.75	78.88 ± 19.55^#^	79.89 ± 16.74
IL-2	122.4 ± 38.41	114.1 ± 19.90	93.48 ± 24.61^#^	89.49 ± 21.78	98.90 ± 29.66^#^	97.82 ± 28.72
IL-6	87.90 ± 32.24	91.03 ± 26.33	69.37 ± 9.38^#^	71.81 ± 18.29	66.75 ± 19.22^#^	64.72 ± 18.39
IL-8	271.96 ± 86.43	272.8 ± 74.72	141.75 ± 28.57^#^	147.11 ± 23.97	146.1 ± 33.22^#^	148.0 ± 30.27

^#^*P* < 0.05 compared with NC

## Discussion

The current study screened the expression profile of TLRs in CD8^+^ T cells, showing a robust down-regulation of TLR2 in GC patients. Furthermore, peripheral and tumor-infiltrating CD8^+^ T cells presented exhausted property in GC patients. Importantly, activation of TLR2 only induced cytolytic activtity, but not non-cytolytic function, of peripheral and tumor-infiltrating CD8^+^ T cells from GC patients. This process was accompanied by enhancing perforin and granzyme B, without influencing co-inhibitory checkpoints expression or proinflammatory cytokines production, in CD8^+^ T cells. The present data suggested a direct immunoregulatory activity of TLR2 signal to CD8^+^ T cells, and indicated that restrained TLR2 might be insufficient to induce functional cytotoxicity of CD8^+^ T cells, which was probably associated with tumor cells evasion and metastasis in GC patients.

TLRs signaling revealed dual function in tumorigenesis due to the potential context-dependent antitumor and protumor nature of TLRs activation [9]. Diakowska et al*.* revealed differences in the expression patterns of TLRs between esophageal squamous cell carcinoma and gasrtoesophageal junction adenocarcinoma, and elevated level of serum TLR4 was reported as a potential marker of gastro-esophageal junction cancer [22]. In contrast, Kasurinen et al*.* reported that high tissue TLR5 expression predicted a better outcome of GC [23]. However, few studies focused on the expression profile of TLRs within T cells in GC patients. Previous study by Freeman et al. showed the increased expression of TLR2 on lung CD8^+^ T cells in patient with chronic obstructive pulmonary disease [24]. Herein, we screened the expression of TLR family members in CD8^+^ T cells in mRNA and protein level in GC patients, and found that the transcripts corresponding to TLR family members could be detected within CD8^+^ T cells from both healthy controls and GC patients. Significant down-regulations of TLR2 and TLR7 mRNA were found in both peripheral and tumor-infiltrating CD8^+^ T cells from GC patients. However, TLR7 did not show accompanied reduction with mRNA expression in protein level. The difference in mRNA and protein level of TLR7 in GC patients might be due to degradation of mRNA or protein, modification and folding of the protein post translation. Importantly, TLR2 presented decreased trend in protein level, which was in line with mRNA relative expression in CD8^+^ T cells in GC patients. This indicated that TLR2 signal might directly regulate the function of CD8^+^ T cells, facilitating the pathogenesis and progression of GC.

The impairment of functional CD8^+^ T cells was mainly induced in chronic infection and cancers, leading to the immune exhaustion. CD8^+^ T cell exhaustion was also reported in GC patients, which presented as decreased cytotoxic molecules and increased expression of co-inhibitory checkpoints [25, 26]. In this study, we further confirmed that both peripheral and tumor-infiltrating CD8^+^ T cells revealed exhausted phenotype in GC patients. Moreover, the beneficial effect of CD8^+^ T cells in malignant tumors was not only dependent on direct cytolytic activity, but also attributable to non-cytolytic function which mediated by cytokine production [27]. On the one hand, perforin-granzyme signaling and Fas-FasL interaction was two major pathways inducing the cytotoxic activity of CD8^+^ T cells [28]. Importantly, this process required direct cell-to-cell contact and matching of HLA type for in vitro co-culture. Thus, we chose CD8^+^ T cells from HLA-A*0201 restricted individuals for the co-culture experiments with AGS cells. We found that periforin and granzyme B secretion by CD8^+^ T cells and mRNA level in CD8^+^ T cells, but not FasL expression, was robustly down-regulated in GC patients. The co-inhibitory checkpoint PD-1, but not LAG-3 expression, was elevated in CD8^+^ T cells from GC patients. Furthermore, target cell death, which induced by peripheral and tumor-infiltrating CD8^+^ T cells from GC patients in direct contact manner, was also suppressed. On the other hand, proinflammatory cytokine secretion, no matter in contact coculture system or in solely cultured system, was robustly reduced in GC derived-CD8^+^ T cells. However, the cytotoxic of CD8^+^ T cells in indirect contact coculture system was comparable between healthy controls and GC patients. This suggested that proinflammatory cytokines production was not effective for tumor rejection even in the healthy situations. Thus, GC induced CD8^+^ T cells exhaustion might be mainly due to the dysfunction in cytolytic activity probably via suppression of perforin-granzyme B pathway and elevation of PD-1 expression.

TLR2 co-stimulation was more responsible for proliferation and survival of CD8^+^ T cells rather than for CD4^+^ T cells [29]. TLR2 engagement on CD8^+^ T cells not only enabled generation of functional memorial cytotoxic T cells response even in a suboptimal T cell receptor stimulation [30, 31], but also directly contributed to the maintenance of diversity of antigen-specific CD8^+^ T cells in response to microbial stimuli or endogenous danger signals [32]. Importantly, TLR2 co-stimulation drive polyfunctional CD8^+^ T cell responses [33]. Thus, TLR2 activation became a potential hallmark for reconstruction of CD8^+^ T cell activity for treatment of viral infection and cancer [34]. TLR2 pre-activation promoted CD8^+^ T cell responses and accelerated viral clearance in hepatitis B virus infected mouse models [35]. Tumor-resident TLR2 also essential for the tumor-triggered elevation of major histocompatibility complex class I, which not only increased antigen presentation but also contributed to the proliferation and activation of CD8^+^ T cells [36, 37]. We showed that activation of TLR2 by Pam3Csk4 mainly enhanced perforin and granzyme B secretion by CD8^+^ T cells from both healthy controls and GC patients, however, only promoted cytotoxicity in GC. The less reaction of CD8^+^ T cells to TLR2 ligands in healthy controls might due to the following two reasons. Firstly, we only enrolled six HLA-A*0201 restricted healthy controls, which required enlargement of sample size to confirm the result. Secondly, CD8^+^ T cells from healthy controls did not pre-sensitized with tumor antigens. The number of tumor-antigen specific CD8^+^ T cells was limited to perform direct cytolytic function. Moreover, TLR2 stimulation did not affect the cytokine production or co-inhibitory molecules by CD8^+^ T cells. Thus, the current results indicated TLR2 activation induced CD8^+^ T cell response in GC patients might be mainly based on promotion of perforin-granzyme pathway. Further experiments were also needed to confirm the TLR2 regulation to CD8^+^ T cells in vivo and to analyze the tumor antigen specific CD8^+^ T cell response to TLR2 activation in the pathogenesis of GC.

## Conclusion

In summary, GC induced peripheral and tumor-infiltrating CD8^+^ T cells exhaustion, which mainly presented as dysfunctional cytolytic activity. Down-regulation of TLR2 in CD8^+^ T cells might contribute to immune dysfunction of CD8^+^ T cells probably via suppression of perforin-granzyme pathway. TLR2 activation might be considered as one of the therapeutic targets for GC treatment.

## Data Availability

The datasets used and analyzed in the current study are available from the corresponding author on reasonable request.

## Abbreviations

ELISA: Enzyme-linked immunosorbent assay
ELISPOT: Enzyme-linked immunospot assay
FBS: Fetal bovine serum
GC: Gastric cancer
IFN-γ: Interferon-γ
IL: Interleukin
LAG-3: Lymphocyte activation gene-3
LDH: Lactate dehydrogenase
NC: Normal control
PBMC: Peripheral blood mononuclear cells
PCR: Polymerase chain reaction
PD-1: Programmed death-1
SD: Standard deviation
SFC: Spot-form cells
TIL: Tumor-infiltrating lymphocytes
TLRs: Toll-like receptors
TNF-α: Tumor necrosis factor-α
